# Update on FXR Biology: Promising Therapeutic Target?

**DOI:** 10.3390/ijms19072069

**Published:** 2018-07-16

**Authors:** Chang Yeob Han

**Affiliations:** Department of Pharmacology, School of Medicine, Wonkwang University, 460 Iksandae-ro, Iksan 54538, Jeonbuk, Korea; hancy17@wku.ac.kr; Tel.: +82-63-850-6776

**Keywords:** nuclear receptor, FXR (farnesoid X receptor), liver diseases, metabolic disorders, pharmacological application

## Abstract

Farnesoid X receptor (FXR), a metabolic nuclear receptor, plays critical roles in the maintenance of systemic energy homeostasis and the integrity of many organs, including liver and intestine. It regulates bile acid, lipid, and glucose metabolism, and contributes to inter-organ communication, in particular the enterohepatic signaling pathway, through bile acids and fibroblast growth factor-15/19 (FGF-15/19). The metabolic effects of FXR are also involved in gut microbiota. In addition, FXR has various functions in the kidney, adipose tissue, pancreas, cardiovascular system, and tumorigenesis. Consequently, the deregulation of FXR may lead to abnormalities of specific organs and metabolic dysfunction, allowing the protein as an attractive therapeutic target for the management of liver and/or metabolic diseases. Indeed, many FXR agonists have been being developed and are under pre-clinical and clinical investigations. Although obeticholic acid (OCA) is one of the promising candidates, significant safety issues have remained. The effects of FXR modulation might be multifaceted according to tissue specificity, disease type, and/or energy status, suggesting the careful use of FXR agonists. This review summarizes the current knowledge of systemic FXR biology in various organs and the gut–liver axis, particularly regarding the recent advancement in these fields, and also provides pharmacological aspects of FXR modulation for rational therapeutic strategies and novel drug development.

## 1. Introduction

Farnesoid X receptor (FXR; NR1H4), a member of the nuclear receptor (NR) superfamily, was identified as a receptor of bile acids (BAs) [1,2,3]. As a ligand-activated transcription factor, FXR binds to DNA (i.e., FXR response elements) as a monomer or as a heterodimer with a common partner for NRs, retinoid X receptor (RXR; NR2B1) [4,5], in order to regulate the expression of the diverse genes involved in the metabolism of BAs, lipids, and carbohydrates [5,6]. There are two known *FXR* genes (i.e., *FXRα* (NR1H4) and *FXRβ* (*NR1H5*)). A single *FXR*α gene in human and rodents encodes four different isoforms (*FXRα1*, *FXRα2*, *FXRα3*, and *FXRα4*), resulting from different promoters and RNA splicing [5,7]. *FXRα* is evolutionally conserved across species, from fish to humans, whereas the functional role of *FXRβ*, a pseudogene in human and primates, remains unclear [5,6]. FXR is mainly expressed in various tissues including the liver, intestine, kidney, and adrenal gland, with much lesser extents in the adipose tissue and heart [5,7]. Given the tissue distribution and biological functions of FXR, it serves as a key metabolic regulator of systemic energy homeostasis.

FXR plays crucial roles in the regulation of BA synthesis, secretion, and transport; thus, it has been considered as a promising target for the treatment of cholestatic disorders, including primary biliary cirrhosis (PBC) [5,7,8]. FXR is also important for the regulation of the lipid and glucose metabolism [5,7]. The activation of FXR showed beneficial effects on various metabolic diseases, including fatty liver diseases, type 2 diabetes, dyslipidemia, and obesity [5,7,9,10]. Accumulating evidence suggests that FXR agonism is favorable to liver regeneration and hepatocarcinogenesis [11,12], and contributes to the protection of atherosclerosis and renal diseases [13,14], indicating the systemic effects of FXR activation.

During the past decade, there were a lot of efforts to discover and optimize potential drug candidates for the FXR activation. Obeticholic acid (OCA), also known as INT-747 or 6α-ethyl-chenodeoxycholic acid (6-ECDCA), was proposed as a promising therapeutic agent for the treatment of liver and/or metabolic diseases. However, the unwanted effects of FXR activation have been discovered in certain conditions, and the FXR antagonists are also being developed. It is noteworthy that the effects of FXR modulation are multifaceted, probably due to diverse conditions (e.g., tissue specificity, disease type and state, pathologic stimuli, and/or nutrient/energy status). As novel findings on the complexity of FXR and/or BA biology are being provided, it allows us to re-evaluate the value of FXR as a therapeutic target. This review focuses on the recent advancement of FXR biology in various organs and the gut–liver axis, and also discusses the pharmacological consideration of FXR modulators for therapeutic approaches.

## 2. Biological Roles of FXR in Various Organs and the Inter-Organ Network

**Figure 1:**
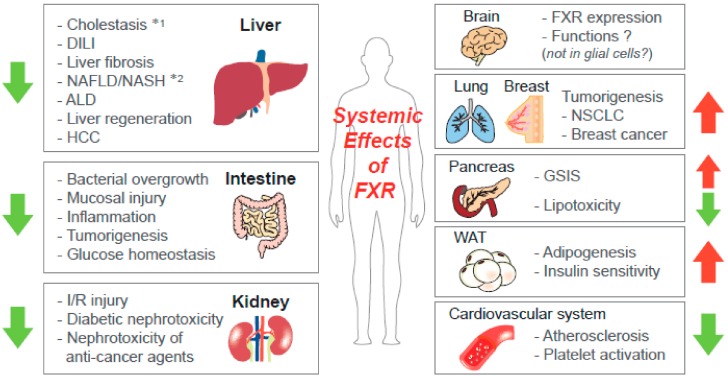
Systemic effects of farnesoid X receptor (FXR) activation. FXR contributes to maintenance of homeostasis and the integrity of various organs, including the liver. The arrows present the effects of FXR agonism on the pathophysiology of the organs (Note: *1—the effects of FXR on non-obstructive and obstructive cholestasis are different; *2—the roles of tissue-specific FXR in metabolic disorders are varying according to conditions). ALD—alcoholic liver disease; DILI—drug-induced liver injury; GSIS—glucose-stimulated insulin secretion; HCC—hepatocellular carcinoma; I/R—ischemia-reperfusion; NAFLD—nonalcoholic fatty liver disease; NASH—nonalcoholic steatohepatitis; NSCLC—non-small cell lung cancer.

The biological functions of FXR are intensively studied in aspects of BA metabolism. The genes involved in BA biosynthesis are suppressed by FXR activation, while those responsible for BA secretion are induced [7]. Following studies using genetic mouse models with a knockout (KO) system demonstrated that FXR controls the lipid and glucose metabolism, which may depend on pathophysiologic conditions; the FXR activation inhibits hyperglycemia and dyslipidemia in metabolic disease states, but it also contributes to normal glucose production in fasting conditions [5]. Besides the actions of FXR as a sensor for the regulation of the whole-body energy metabolism, it has effects on integrity maintenance in various organs, including the liver, intestine, kidney, white adipose tissue (WAT), and pancreas, which might be closely interconncected with metabolic functions (Figure 1).

### 2.1. Functions of FXR in the Liver

Given the abundance of the FXR expression in the liver and the enterohepatic circulation of the BAs, this NR plays a key role in liver pathophysiology. Most of the research for the FXR and/or BAs are directly (i.e., autonomous effects of hepatic FXR) or indirectly (i.e., crosstalk to FXR signaling in other tissues) related to hepatic functions, indicating the importance of FXR biology for understanding the pathogenesis of liver diseases and the associated systemic effects.

#### 2.1.1. Cholestasis

BAs are primarily synthesized by the liver, which is the route of eliminating cholesterol [7]. Although BAs are physiologically important for lipid metabolism and hormonal regulation, the intrahepatic accumulation of cytotoxic BAs can cause liver injury, ultimately leading to biliary fibrosis and cirrhosis [15]. Thus, FXR can protect the liver against the burden of toxic BAs. The expression of bile salt export pump (BSEP) rigorously depends on FXR and is a critical determinant of the cholestatic phenotype and of liver injury; the FXR KO mice with bile duct ligation (BDL) operation exhibited the undetectable hepatic BSEP expression and disseminated hepatocyte necrosis, despite of decreased intraductal pressure and fewer bile infarcts [16]. The expressions of other ATP-binding cassette (ABC) transporters, such as multidrug resistance-associated protein (MRP)3 and MRP4, were induced by the FXR-independent manner [16], thus the canalicular BSEP seems to be one of the key molecules associated with FXR-dependent hepatoprotection upon the BAs loading, which was also demonstrated by a different model with cholic acid and ursodeoxycholic acid (UDCA) treatment [17]. The *BSEP* gene repression was also linked to drug-induced cholestatic liver toxicity [18]. In addition, the expressions of basolateral BA transporters, organic solute transporter (OST)- α/β, were induced by FXR activation and contributed to the coordinated detoxification of BAs in cholestasis [19,20]. FXR transcriptionally regulates the expression of organic anion transporting polypeptide 1B1 (OATP1B1), a transporter responsible for hepatocellular uptake of many endogenous molecules, suggesting the potential roles of FXR in OATP1B1-associated drug interactions, drug-induced liver injury, and cholestasis [21].

The downstream targets and/or upstream regulators of FXR can affect the BA metabolism and cholestasis. The small heterodimer partner (SHP; NR0B2), an atypical orphan NR lacking a DNA-binding domain [22], is a target gene of FXR and accounts for the inhibition of the cholesterol 7α-hydroxylase (*CYP7A1*) gene as the rate-limiting enzyme for BA biosynthesis [23,24], contributing to feedback inhibition of the BAs to maintain homeostasis. Consistently, an SHP deficiency showed the increased expression of CYP7A1 and sensitized the mice to liver injury from obstructive cholestasis [25]. So, the combined loss of FXR and SHP in mice induced CYP7A1 expression along with the increase in the *CYP17A1* gene, resulting in juvenile onset cholestasis and liver injury [26]. β-catenin was recently suggested to bind FXR and inhibit its activity; thus, the loss of β-catenin led to increases in the FXR nuclear translocation and binding to RXR, which decreased the total BAs and hepatic injury [27].

On the other hand, the activation of FXR also has a detrimental effect on obstructive cholestasis [28]. FXR suppresses the ability of the constitutive androstane receptor (CAR) to activate the *MRP4* promoter, supporting that the FXR activation in obstructive cholestasis may aggravate liver injury by inhibiting a protective mechanism elicited by CAR [29]. FXR antagonism by theonellasterol, a 4-methylene-24-ethylsteroid isolated from the marine sponge, Theonella swinhoei, led to the increase in MRP4 expression in the liver, which protected against liver injury in cholestasis [30]. As FXR, pregnane X receptor (PXR), and CAR ligands regulate different target genes, it seems that a combination of ligands/activators of FXR, PXR, and/or CAR could reduce the potential side effects of FXR activation alone in severe cholestasis [31]. The CAR/PXR activation was involved in the different patterns of intrahepatic cholestasis models (i.e., FXR/SHP double KO vs. BSEP KO), suggesting the heterogeneity of intrahepatic cholestasis [32]. Thus, alternative/basolateral overflow along with the renal excretion systems of BAs may be important for reducing the BA accumulation in cholestasis [33].

#### 2.1.2. Drug-Induced Liver Injury (DILI)

DILI, a frequent cause of hepatotoxicity, can develop following the use of a number of drugs and is one of the important clinical problems. Recently, Lu et al. developed a systems pharmacology approach utilizing the integrating network analysis and molecular modeling to explore the molecular mechanisms of DILI, and proposed that FXR antagonism by nonsteroidal anti-inflammatory drugs (e.g., indomethacin and ibuprofen) may contribute to DILI, providing novel insight into the basis of liver injury for the use of the drugs [34]. In addition, the administration of triptolide (a diterpenoid isolated from Tripterygium wilfordii Hook F), having immunosuppressive and anti-tumor activities, decreased the expressions of FXR and the silent information regulator 1 (SIRT1) (a nicotinamide adenine dinucleotide (NAD)-dependent deacetylase regulating FXR activity [35]) in the liver of rats [36]. The liver damages induced by triptolide were diminished with the treatment of a SIRT1 agonist SRT1720 or an FXR agonist OCA, indicating the protective effect of FXR on triptolide-induced hepatotoxicity [36].

#### 2.1.3. Liver Injury and Fibrosis

Liver fibrosis is the state of excessive deposition of the extracellular matrix (ECM) proteins, including collagen, which occurs in most types of chronic liver diseases in response to repeated liver damage. FXR agonists have been shown to offer therapeutic benefits in patients with PBC [37]. The activation of FXR has hepatoprotective effects on various cytotoxic stimuli as well as the BAs overload. In mice fed a methionine and choline-deficient (MCD) diet, as a murine model of nonalcoholic steatohepatitis (NASH), the FXR agonist WAY-362450 (also known as FXR-450) lessened the hepatic inflammation and fibrosis development [38]. The FXR expression was reduced in the fibrotic liver tissues of humans and mice, and the activation of FXR inhibited mitochondrial dysfunction and the subsequent hepatocyte death through the repression of miR-199a-3p targeting liver kinase B1, an upstream kinase of AMP-activated protein kinase (AMPK) [39]. In addition, the AMPK activation by liquiritigenin repressed the oxidative hepatic injury induced by serum deprivation as mediated by the induction of FXR [40]. The loss of FXR resulted in the increase in circulating taurocholate and hepatic c-Jun-N-terminal kinase signaling, inducing susceptibility to carbon tetrachloride (CCl_4_)-induced liver injury [41]. A recent study reported that FXR signaling activated the hepatic inositol-requiring enzyme 1α/X-box binding protein 1 pathway of the unfolded protein response [42], suggesting the possible role of FXR as a therapeutic target for endoplasmic reticulum (ER) stress-associated liver diseases.

The activated hepatic stellate cells (HSCs) are critical for the initiation and progression of liver fibrosis, by producing fibrogenic factors and ECM proteins [43]. Although FXR was reported to be marginally expressed in human HSCs [44], the roles of FXR in HSC biology were demonstrated. The FXR-SHP regulatory cascade mediated the inhibition of HSCs and promoted the resolution of liver fibrosis [45,46]. In addition, the FXR ligands treatment increased the peroxisome proliferator-activated receptor (PPAR)-γ mRNA levels in the HSCs and in the rodent models of liver fibrosis, leading to the inhibition of the HSCs activation [47]. The induction of PPARγ by FXR activation was mediated SHP-dependently and -independently [48]. FXR activation inhibited the endothelin-1-mediated contraction of HSCs [49], and increased the miR-29a promoter activity responsible for the inhibition of ECM production in HSCs [50].

#### 2.1.4. Nonalcoholic Fatty Liver Disease (NAFLD)

NAFLD is the most common event of liver pathogenesis, which is characterized by the accumulation of fat within hepatocytes. It can lead to progressive NASH, fibrosis, and, ultimately, hepatocellular carcinoma (HCC) and liver failure [51]. In NAFLD patients, the decreased expression of hepatic FXR was accompanied by increases in the liver X receptor (LXR), sterol response element binding protein-1c (SREBP-1c), hepatic triglyceride synthesis, and the degree of liver steatosis [52]. In the obese animal model, the FXR activation by the OCA treatment inhibited the development of steatosis [53]. In addition, WAY-362450 attenuated the fructose-induced hepatic steatosis through the suppression of inflammation and the hepatic lipid droplet protein in mice [54]. The FXR activation by OCA treatment, in combination with miR-21 ablation, ameliorated the NASH-associated liver damage in mice [55]. MiR-194 directly binds to FXR 3′-untranslated region, thus, FXR upregulation by the inhibition of miR-194 contributes to the prevention of high-fat diet (HFD)-induced hepatic steatosis in mice [56]. The serum BA levels correlated with the disease severity in NAFLD, whereas the adiponectin levels were inversely correlated [57], supporting that treatment with FXR agonists and/or adiponectin activators may be of help for the prevention of NASH.

On the other hand, the global double KO of FXR/SHP reduced adiposity and showed beneficial effects on glucose and lipid metabolism in the aged mice, despite inducing cholestasis and liver injury at early ages [58]; the liver-specific FXR/SHP double KO mice exhibited similar phenotypes. In both global and liver-specific FXR/SHP double KO livers, the metabolic alterations were associated with the changed expression of the fatty acid metabolism and autophagy genes, which are responsible for fatty acid usage. Consistently, the liver-specific SHP deletion protected against hepatic steatosis development by repressing the expression of PPARγ2 and the lipid-droplet protein fat-specific protein 27 β [59]. These results indicate that precise understanding of FXR and its downstream targets is a prerequisite for the management of metabolic liver diseases.

A recent study showed that FXR regulates hepatic amino acid catabolism and the detoxification of ammonium through ureagenesis and glutamine synthesis in mice [60]; FXR activation by OCA treatment increased the expressions of genes involved in amino acid degradation, ureagenesis, and glutamine synthesis, whereas the loss of FXR had opposite effects, suggesting the novel function of FXR in the regulation of hyperammonemia as a common complication in patients with acute and chronic liver diseases.

#### 2.1.5. Alcoholic Fatty Liver Disease

Alcoholic liver disease (ALD) is one of the main complications of alcohol abuse. The effects of several compounds affecting FXR signaling were examined in animal models with ALD. The FXR activity was reduced in the liver of the mice that were fed a Lieber-DeCarli ethanol diet, and the pharmacological activation of FXR by WAY-362450 diminished the oxidative liver injury [61]. Consistently, the loss of FXR lessened the forkhead box O3a (FOXO3a)-mediated autophagy, which exaggerated the alcohol-induced liver injury [62]. In addition, the hepatic BA accumulation activated nuclear factor kappa B and promoted the progression of alcoholic steatohepatitis in FXR KO mice, which was attenuated by UDCA treatment [63]. Curcumin was suggested to decrease alcohol-induced hepatic steatosis via the regulation of nuclear factor erythroid-derived 2-related factor 2 (Nrf2)/FXR signaling in hepatocytes [64]. The therapeutic effects of the FXR activation on ALD need to be further validated in patients.

#### 2.1.6. Liver Regeneration

FXR plays a role in liver regeneration, which is an important function of the organ in order to repair injury [6,65]. Huang el al. demonstrated that BA signaling is required for normal liver regeneration, and FXR activation by increased BA flux might be a signal of a reduced functional capacity of the liver [66]. Upon CCl_4_-induced toxic injury, the FXR-null mice had more severe defects in liver repair than the wild-type mice, which was accompanied by a higher mortality and increased hepatocyte death [67]. The FXR activation promoted hepatocyte proliferation through the gene induction of pyruvate dehydrogenate kinase 4 (*PDK4*) as well as the subsequent metabolic reprogramming for rapid biomass generation, linking between liver regeneration and metabolic switch [68]. As a regulator of FXR, SIRT1 is also involved in liver regeneration. The SIRT1 transgenic mice that overexpress SIRT1 exhibited BA accumulation, weakened hepatocyte proliferation, and augmented mortality after partial hepatectomy surgery [69]. The effects of SIRT deregulation seem to be associated with the impaired FXR activity due to persistent deacetylation [69]. Collectively, FXR activation promotes liver repair after injury, and the FXR-mediated BA signaling might be an integrated factor of normal regeneration, indicating the value of FXR activators to modulate liver regeneration after hepatic transplantation or the resection of liver cancer [65].

#### 2.1.7. HCC

In the absence of FXR, liver cancers are spontaneously developed in mice, which is accompanied with prominent hepatic injury and inflammation [70]. Lowering the BA pool in the FXR KO mice by feeding 2% cholestyramine, significantly inhibited the tumor lesions, suggesting the link between metabolic regulation and liver carcinogenesis [70]. The upregulation of interferon gamma (IFNγ) in the liver of FXR-deficient mice suppresses carcinogenesis by inducing p53 expression and avoiding the activation of signal transducer and activator of transcription 3 (STAT3) [71]; thus, IFNγ may act as a protective mechanism against hepatocarcinogenesis in association with FXR inactivation. The enforced expression of SHP partially protected against liver cancer development in the FXR null mice [72]; hepatocyte-specific SHP overexpression showed a lower grade of dysplasia and reduced cell proliferation in the FXR KO mice, although the incidence or size of the liver tumor was not affected, suggesting the possibilities of FXR-dependent but SHP-independent hepatic tumorigenesis. Chronic infection with hepatitis B virus is one of the major risk factors for HCC. Recently, it has been proposed that the loss of FXR facilitates hepatitis B virus X protein (HBx)-induced liver cancer development in mice [73,74]; full-length HBx, but not C-terminally truncated variants of HBx, increased the FXR transactivation, thus the FXR-HBx interaction may exert a protective mechanism to inhibit HCC. FXR ablation also enhanced BA levels and the development of HCC induced by the deregulation of the circadian rhythm [75]. The interruption of hepatic stem cell differentiation into hepatocytes is related to hepatoblastoma development. FXR repressed the action of gankyrin oncogene to induce the differentiation of hepatic stem cells, which was mediated by tumor suppressor proteins, including p53 [76].

#### 2.1.8. Inflammation

FXR has anti-inflammatory effects in the liver. Both natural ligand CDCA and synthetic agonist GW4064 raised the expression of the suppressor of cytokine signaling 3, a negative regulator of cytokine-STAT3 signaling, contributing to the protection of hepatocellular inflammation [77]. In addition, the FXR activation also lessened apoptosis and liver injury in concanavalin A-induced autoimmune hepatitis in mice [78]. A recent study showed that FXR activation by OCA treatment ameliorated the HFD-induced hepatic inflammation through the reprogramming of arachidonate metabolism, by inducing a CYP450 epoxygenase expression [79]. In addition, the GW4064 treatment inhibited Toll-like receptor 4 (TLR4)-mediated p38 mitogen-activated protein kinase signaling pathway in mice, leading to the prevention of lipopolysaccharide-induced hepatic inflammation [80]. The amplified plasma levels of BAs are important for the prediction of sepsis-associated mortality, and cholestasis is a common complication of sepsis. It has been suggested that FXR involves in the regulation of cholestasis-associated sepsis, which can be mediated by the negative regulation of NACHT, leucin-rich-repeat (LRR), and pyrin (PYD) domains-containing protein 3 (NLRP3) inflammasome via the direct binding of FXR to NLRP3 and caspase 1 in macrophages [81,82]; thus, the FXR deficiency sensitized the mice to endotoxemia shock. Furthermore, the GW4064 inhibited the NLRP3 inflammasome activation independent of the FXR activation in macrophages [83]. These results suggest the potential roles of FXR agonism in the treatment of NLRP3-related diseases. The effects of FXR on inflammatory features and inflammasome activation in liver parenchymal cells need investigations.

### 2.2. Functions of FXR in Non-Hepatic Organs

#### 2.2.1. Renal Function

FXR has a key role in protecting against kidney injury induced by various toxic stimuli. FXR activation modulated the renal expressions of lipid metabolism genes, profibrotic growth factors, and proinflammatory cytokines, leading to the inhibition of diabetic nephrotoxicity [84]. Recently, it has been shown that chemical chaperone tauroursodeoxycholic acid diminished maladaptive ER stress signaling through FXR activation, specifically in tubular cells, and reduced tubular injury in leptin receptor-deficient diabetic (db/db) mice [85]. In addition, the FXR activation also protected the kidney from ischemia-reperfusion (I/R) damage [86]; I/R induced pathologic changes (i.e., increased creatinine, albuminuria, tubular necrosis, and apoptosis) were prohibited and the renal function was improved in the mice pretreated with OCA. Moreover, the OCA treatment prevented cisplatin-induced kidney injury by enhancing SHP, which might be related to anti-fibrotic, anti-inflammatory, and anti-apoptotic effects [87]. Dioscin directly targeted the FXR, and subsequently suppressed the doxorubicin-induced nephrotoxicity in rats [88]. FXR activation contributed to the maintenance of glutathione homeostasis and inhibited kidney damage in the uninephrectomized obese mice [89]. The FXR inhibition of renal fibrosis was mediated by the suppression of the Smad3 expression in the mice with the unilateral ureteral obstruction model [90]. The chromatin immunoprecipitation experiments revealed the binding of hepatocyte nuclear factor (HNF)-1β to *FXR* promoter in the kidney of mice, suggesting the involvement of FXR in the HNF-1β-mediated regulation of urinary concentration and response to hypertonicity [91]. Consistently, under hypertonic stress, FXR seems to be important for the viability of renal medullary collecting duct cells via inducing tonicity response enhancer binding protein expression and its nuclear translocation [92].

#### 2.2.2. Intestinal Function

Biliary obstruction can cause bacterial proliferation, which leads to the translocation of bacteria across the mucosal barrier and systemic infection. FXR raised the expression of genes involved in enteroprotection, and impeded bacterial overgrowth and mucosal injury in ileum of the mice operated with BDL [93]. FXR activation also protected against intestinal tumorigenesis through the induction of apoptosis and elimination of genetically altered cells [94]. In addition, FXR activation by OCA treatment inhibited the chemically induced intestinal inflammation in mice, proposing the role of FXR in the intestinal epithelial barrier in inflammatory bowel disease [95].

#### 2.2.3. Function in WAT

FXR promotes adipogenesis and peripheral insulin sensitivity [96]; the FXR-null mice exhibited a reduced mass of adipose tissue and lower concentrations of serum leptin. FXR expression was induced during preadipocyte differentiation in vitro and expressed at a low level in WAT in vivo. The exposure of differentiated 3T3-L1 adipocytes to GW4064 enhanced insulin-induced signaling and glucose uptake. The GW4064 treatment also inhibited insulin resistance in genetically obese leptin-deficient (ob/ob) mice [96]. Consistently, the treatment of 3T3-L1 cells with OCA increased the adipocyte differentiation, which was associated with the induction of aP2, CCAAT-enhancer-binding protein (C/EBP)-α, and PPARγ2 transcripts together with other adipocyte-related genes [97]. In addition, the FXR activation by CDCA repressed several pro-inflammatory adipokines (e.g., tumor necrosis factor-α, monocyte chemoattractant protein-1, and interleukin-6), and increased anti-inflammatory and insulin sensitizing adipokines (e.g., adiponectin, leptin) in palmitate-treated 3T3-L1 cells and adipose tissues of the HFD-fed rats [98].

#### 2.2.4. Function in Pancreas

FXR induces glucose-stimulated insulin transcription and secretion, which is partly mediated by the induction of the glucose regulated transcription factor KLF11 [99]. FXR is functionally expressed in human islets and β-cell lines, and protects the islets from lipotoxicity [100]. In the FXR KO mice, glucose-stimulated insulin secretion (GSIS) is impaired in the islets despite a normal β-cell mass [100]. It seems that the BA-mediated FXR activation also induces GSIS by non-genomic effects on stimulus-secretion coupling [101]. In contrast to the lean mice, GW4064 had no effect on the insulin secretion of islets from mice fed a HFD, indicating that the FXR signaling in β-cells is dysregulated under an overnutrition condition [102]. However, the FXR deficiency did not significantly affect acute pancreatitis in mice [103], suggesting that further investigation will be needed to understand the pathophysiological roles of FXR in the pancreas.

#### 2.2.5. Cardiovascular Function

FXR has been also been suggested as a therapeutic target for atherosclerosis [13]; FXR agonism has protective effects on the development and progression of atherosclerosis, while simultaneous side effects, including the reduction of plasma high-density lipoprotein, have been reported. A recent study showed that FXR activation by GW4064 diminished the myocardial infarction-induced cardiac remodeling and dysfunction, by stimulating the adiponectin secretion in mice [104]. Despite the lack of nucleus, the platelets have various transcription factors that regulate their function via DNA-independent manners. The activation of platelets was repressed by FXR ligands, such as GW4064 and OCA, in response to the stimulation of collagen or thrombin receptors, which led to the reduction of calcium mobilization, fibrinogen binding, and aggregation [105]. The inhibitory effects were not observed in the FXR-deficient platelets, indicating the possible nongenomic actions of FXR for the regulation of platelet functions. The ability of the FXR ligands to inhibit platelet activation and thrombus formation was also mediated by the initiation of coated platelet formation and consequent desensitization to platelet stimuli through the inhibition of integrin αIIβb3 [106].

#### 2.2.6. Effects on Tumorigenesis

Although FXR has the anti-tumor effects on hepatic and intestinal cancers, the impacts of FXR on the carcinogenesis of other tissues seem to be different. In breast carcinoma patients, the expression levels of FXR were positively correlated with tumor size and the proliferative rate of tumor cells [107], suggesting FXR as a prognosticator of invasive breast carcinoma. Consistently, the FXR expression was significantly increased in non-small cell lung cancer (NSCLC), which stimulated tumor growth through the direct transactivation of the cyclin D1 (*CCND1*) gene [108]. Therefore, FXR may have pleiotropic effects on tumorigenesis, according to tissue types; FXR may primarily act as a tumor suppressor gene in enterohepatic tissues, but it can be a proto-oncogene in other tissues, including breast and lung.

#### 2.2.7. Neuronal Function

Recently, FXR mRNA and protein expression were identified in mouse brain neurons and in mouse/human brain tissues [109]; FXR was predominantly localized in the nucleus in cultured neurons, whereas it was mainly found in the cytoplasm in the neurons in vivo. The activation of FXR augmented the SHP expression levels in cultured neurons and in brain tissues, suggesting the potential functional roles of FXR in brain neurons. However, the functions of the brain-resident glial cells, such as oligodendrocytes, astrocytes, or microglia, were not altered by FXR activation [110]. So, further investigations will be needed to elucidate the pathophysiological roles of FXR in normal brain function and neuronal diseases.

### 2.3. Inter-Organ Function of FXR in the Gut-Liver Axis

**Figure 2:**
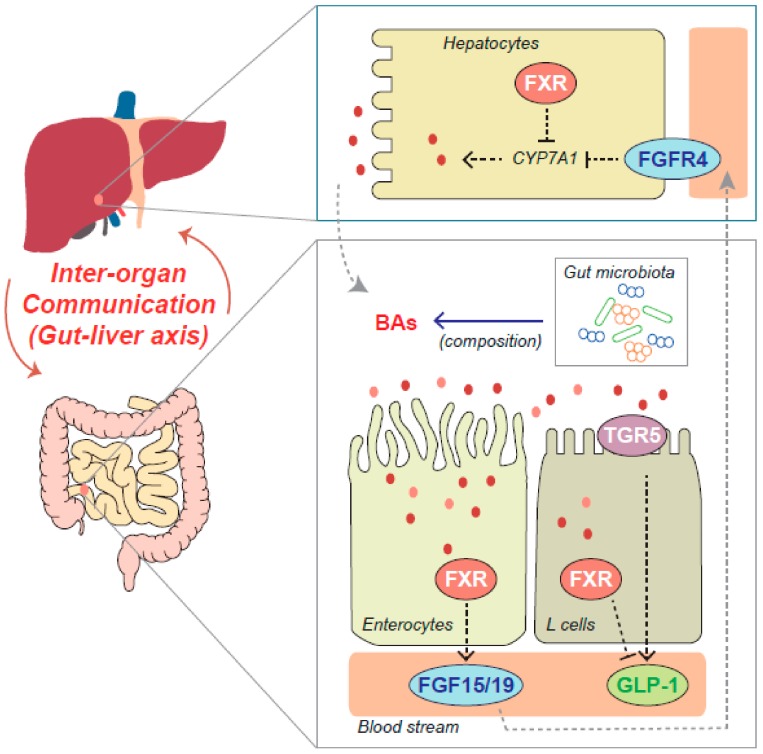
The roles of FXR in the gut–liver axis. FXR contributes to the crosstalk between liver and intestine through the regulation of hepatic bile acids (BAs) synthesis and intestinal fibroblast growth factor-15/19 (FGF-15/19) secretion. FGF15/19 controls the BA production via hepatic FGF receptor 4 (FGFR4) signaling. BAs act on TGR5 as well as FXR, contributing to metabolic homeostasis. Intestinal microbiota affects the composition of BAs, leading to changes in FXR activities. Dotted gray arrows represent the transport of BAs or FGF15/19 through the bile duct or the portal vein, respectively. Dotted black arrows indicate the activating effects, whereas dotted T bar arrows indicate the inhibitory effects. Solid blue arrow represents the effects of gut microbiota on the composition of BAs, while red arrows represent the inter-organ crosstalk between liver and intestine.

Functional communications between the organs are important for the maintenance of systemic homeostasis in the whole organism. Thus, the deregulations of inter-organ crosstalk are closely associated with the pathogenesis of diverse diseases, including metabolic syndrome. In particular, FXR may play a main role in the inter-organ network, as it is involved in the regulation of enterohepatic circulation of BAs. Emerging evidence also proposed the critical roles of fibroblast growth factor (FGF)15/19 (i.e., mouse FGF15 is the ortholog of human FGF19) as a gut-derived peptide hormone mediating the systemic effects of FXR and/or BAs (Figure 2).

FGF15 expression is induced by FXR activation in the small intestine, and it suppresses the expression of CYP7A1 in the liver via the actions of FGF receptor 4 (FGFR4), functioning as a component of a gut–liver signaling pathway to control BA homeostasis [111]. In the study using transgenic mice that express a constitutively active FXR in the intestine, the selective activation of FXR in the intestine protected the liver from cholestasis by increasing the FGF15 expression and decreasing the hepatic BA pool [112]. In addition, the intestinal-specific FXR reactivation was sufficient to normalize BA enterohepatic circulation through FGF15 actions and avoid the development of HCC in FXR KO mice [113]. Intestinal FXR was activated to induce the expression of FGF15 in the intestine in response to liver injury, and the enforced expression of FGF15 recovered the defective liver regeneration and repair in the intestine-specific FXR-deficient mice [114], suggesting the role of FGF15 signals stimulated by intestinal FXR in endocrine system for liver repair processes besides the autonomous effect of hepatic FXR. Moreover, the ileal FGF15 pathway was partly involved in the beneficial effects of the SIRT1 activator, SRT1720, on the inhibition of cholestatic liver injury in mice [115]. Intestinal FXR controlled transintestinal cholesterol excretion via induction of FGF15 for the maintenance of cholesterol homeostasis in mice [116].

The intestinal FXR and FGF15/19 pathway are also closely associated with metabolic disorders. In the NAFLD condition, the proportion of BAs antagonizing the FXR activity increased, which may account for, at least in part, the reduction of FGF15/19 and FGFR4-mediated signaling [117]. Intestinal FXR agonism, by treatment with a gut-restricted FXR agonist fexaramine, reduced the diet-induced obesity and insulin resistance, and promoted the browning of WAT, accompanying a drastic increase in the enteric FGF15 levels [118]. The BAs activate both the FXR and G protein-coupled BA receptor-1 (Takeda G protein-coupled receptor-5 [TGR5]) to control BA homeostasis and glucose metabolism. The fexaramine-activated intestinal FXR shaped the gut microbiota to activate TGR5 and glucagon-like peptide-1 (GLP-1) signaling to improve hepatic insulin sensitivity and induce adipose tissue browning [119]. FXR induced the TGR5 gene expression to stimulate GLP-1 production and improve hepatic glucose and lipid metabolism in the HFD-induced obese mice [120].

On the other hand, the intestine-selective inhibition of FXR by treatment with glycine-β-muricholic acid, improved HFD-induced and genetic obesity, insulin resistance, and liver steatosis in the mice [121]. The intestinal FXR signals also promoted NAFLD [122]; the mice treated with antibiotics or tempol showed the increases in the conjugated BA metabolites that inhibited intestinal FXR signaling. Intestine-specific FXR disruption reduced the hepatic triglyceride accumulation in HFD-fed mice, which was mainly due to the decrease in circulating ceramides. Consistently, the intestinal FXR-ceramide signaling axis also induced the hepatic gluconeogenesis in mice [123,124]. In addition, the FXR repressed GLP-1 secretion by enteroendocrine L cells [125]; the treatment of ob/ob mice with a BA sequestrant improved the GLP-1 production and hyperglycemia in an FXR-dependent manner. Furthermore, the gut microbiota promoted HFD-induced obesity and associated metabolic dysfunction through FXR [126], indicating the role of FXR in the link between microbiota composition and metabolic diseases. The changes in the intestinal microbiota during the chronic ethanol feeding in mice were associated with the decrease in FXR-FGF15 pathway in enterocytes, and the restoration of the intestinal FXR activity by fexaramine treatment protected the mice from alcohol-induced liver injury [127]. Collectively, these results suggest that the effects of tissue-selective FXR modulation on systemic metabolic homeostasis might be multifaceted, thus the effects of therapeutic approaches targeting FXR need to be carefully interpreted.

## 3. Pharmacological Aspects of FXR Modulation

Based on the biological roles of FXR in BA metabolism and whole-body energy homeostasis, many attempts have been made to develop therapeutic drugs targeting FXR. Indeed, OCA has been investigated in phase II and phase III clinical studies, and the consequences indicated that FXR agonism can be potentially helpful in the management of diseases such as cholestatic liver diseases and metabolic syndrome [128]. WAY-362450 was evaluated in phase I studies in healthy subjects (ClinicalTrials.gov: NCT00499629), but further development was not achieved. GW4064 is one of the most widely used FXR agonists to examine the pharmacological effects of FXR in various cell and/or animal models, but it has not been tested in clinical trials. Several candidate compounds to modulate the FXR activity were also newly identified in pre-clinical studies.

### 3.1. OCA

OCA is a prototype of FXR agonists, which is based on the BA scaffold of CDCA as the most potent endogenous ligand of FXR. Among the FXR agonists tested in clinical studies, OCA has been considered as a promising drug and is shown to have beneficial effects on various disease conditions, as described in the Section 2 (see ‘Biological Roles of FXR in Various Organs and the Inter-Organ Network’). OCA was approved by the Food and Drug Administration for the treatment of patients with PBC, and the clinical study for NASH was recently completed (NCT01265498). Additional clinical trials on alcoholic hepatitis, lipodystropy, or NASH with fibrosis are currently recruiting (NCT02039219, NCT02430077, and NCT02548351). However, there are limitations of common side effects, such as pruritus and gastrointestinal problems, which are often related to its steroidal BA like chemical structure.

### 3.2. INT-767

Recently, a semisynthetic BA derivative INT-767 as a dual FXR and TGR5 agonist was developed [129], and it has been shown that this compound has the ability to inhibit liver injury and metabolic disorders. The INT-767 treatment significantly inhibited liver injury and inflammation, and biliary fibrosis in the MDR2 KO mice, while the FXR-specific OCA and TGR5-specific INT-777 had no protective effects [130]. The dual agonism reduced the cholangiopathy by decreasing the biliary BA secretion and promoting the biliary HCO_3_ output. However, the expression levels of FXR were downregulated, whereas those of the TGR5 were upregulated in the liver of the patients with cholangiocarcinoma [131]. The FXR activation by OCA treatment inhibited, but the TGR5 activation by INT-777 may stimulate, the cholangiocarcinoma progression through the control of cell proliferation, migration, and mitochondrial energy metabolism, suggesting the differential effects of FXR or TGR5 activation on cholangiocarcinoma progression. INT-767 improved the NASH histopathological features in a diet-induced ob/ob mouse model, which showed greater therapeutic potency and efficacy than the OCA treatment [132]. Consistently, INT-767 significantly improved insulin resistance in visceral adipose tissue, and induced the browning of WAT and mitochondrial function [133]. INT-767 also reduced hypercholesterolemia by FXR activation and induced thermogenic gene expression through TGR5 and/or FXR activation, which led to the reversal of the HFD-induced metabolic disorders [134]. Moreover, this dual FXR/TGR5 agonist improved the nephropathy (e.g., proteinuria, podocyte injury, mesangial expansion, and tubulointerstitial fibrosis) in the mouse models with diabetes and obesity [135], and reversed the age-related kidney disease (e.g., proteinuria, podocyte injury, fibronectin accumulation, transforming growth factor (TGF)-β expression, impairments of mitochondrial biogenesis, and function) in mice [136]. These results designate that the dual modulation of FXR and TGR5 could be an attractive therapeutic strategy.

### 3.3. Novel Candidate Compounds for FXR Modulation

Despite the therapeutic advantages of OCA, significant safety issues have still remained, and the problems might be applied to other compounds of a similar structure [137]. Many other FXR agonists are being developed, which are natural and synthetic ligands, including BA derivatives and nonsteroidal compounds, in conjunction with their in vitro/in vivo efficacy and therapeutic applications [9]. Some candidate compounds to directly or indirectly activate FXR activity have been discovered, including natural products, active metabolites, and steroidal/nonsteroidal synthetic compounds, which were almost investigated in pre-clinical studies (Table 1).

On the other hand, it has been suggested that the inhibition of FXR may account for the beneficial effects under certain conditions, as also described in Section 2 (see ‘Biological Roles of FXR in Various Organs and the Inter-Organ Network’). Although the information is not enough yet, the effects of several FXR antagonists on lipid and glucose metabolism were reported in mouse models (Table 2).

### 3.4. Considerations for FXR Modulation

The systemic expression and functions of FXR should be considered for the use of various FXR agonists and the drug development of novel FXR modulators. In addition, the FXR regulation showed multifaceted effects on metabolic disorders and tumorigenesis, possibly due to the differences of tissue, disease state, energy status, and/or experimental conditions [159,160]. FXR has distinct features to regulate energy and nutrient metabolism in postprandial, post-absorptive, and fasting/starvation states [160], suggesting the role of FXR as a fine-tuner or homeostat for energy homeostasis.

As a key mechanism of enterohepatic crosstalk, FGF15/19 affects the systemic energy metabolism. A recent study suggested that mouse FGF15 and human FGF19 exhibited different biological activities in mice; unlike FGF19, FGF15 lacked the protective effects on diabetes remission and did not induce HCC development in the mouse models with metabolic disorders (i.e., db/db, diet-induced obese, and MDR2 KO mice), while both FGF15 and FGF19 suppressed BA synthesis, raising the concern of depending on rodent models for the safety assessment of FXR activators [161].

In another aspect, the endogenous BA ligands of FXR and some synthetic agonists of FXR can also bind to another BA cell surface receptor, TGR5. Dual agonists of FXR and TGR5 seem to be more effective than the agonists of FXR or TGR5 alone, in certain conditions. However, FXR and TGR5 also have different effects on hepatobiliary diseases, indicating that the rational modulation of FXR and/or TGR5 according to situations will be required.

The implications of microbiota and BAs are important for understanding the pathophysiological roles of FXR more in depth, as the intestinal environment is rich in microorganisms and metabolites produced from both the host and colonizing bacteria [162]. Moreover, the gender differences in the BAs and microbiota in association with the actions of FXR also need to be considered for gender dissimilarity in metabolism and metabolic disorders [163].

## 4. Conclusions

FXR plays critical roles in the maintenance of energy homeostasis and the integrity of diverse organs. FXR also contributes to the inter-organ communication, in particular through the enterohepatic signaling pathway (i.e., gut–liver axis). Thus, FXR has been identified as a promising therapeutic target for the management of liver and/or metabolic diseases, allowing the pharmacological approaches to develop FXR modulators. However, FXR has systemic effects on various tissues in the whole body, and the effects of FXR are different according to tissue specificity, disease type, and/or energy status, suggesting the careful use of FXR agonists. Overall, FXR is still an attractive target, but the diversity of FXR biology and several pharmacological aspects of FXR modulation should be of concern for the rational therapeutic strategy and novel drug development.

## Figures and Tables

**Table 1:** Novel candidate compounds for farnesoid X receptor (FXR) agonism/activation. ANIT—α-naphthylisothiocyanate; BA—bile acid; BSEP—bile salt export pump; CAR—constitutive androstane receptor; ER—estrogen receptor; GR—glucocorticoid receptor; HFD—high-fat diet; HSC—hepatic stellate cells; LXR—liver X receptor; NASH—nonalcoholic steatohepatitis; PPAR—peroxisome proliferator-activated receptor; RAR—retinoic acid receptor; ROR—RAR-related orphan receptor; SHP—small heterodimer partner; TGF—transforming growth factor.

Study Type	Compound	Chemical Properties	Proposed Mode of Actions (MOAs) for FXR Modulation	Biological Effects Potentially through FXR Modulation	References
Preclinical	Alisol B 23-acetate (AB23A)	Natural triterpenoid	Direct interaction (molecular docking analysis)	- Promotes liver regeneration in mice after partial hepatectomy - Protects against ANIT-induced hepatotoxity and cholestasis in mice - Protects against CCl_4_-induced hepatotoxicity in mice - Protects against estrogen-induced cholestatic liver injury in mice - Prevents methionine and choline-deficient diet-induced NASH in mice Note: also suggested to have ligand binding activity of PXR, but not CAR, LXR, FXR, PPARα, PPARδ and PPARγ [138]	[139,140,141,142,143]
Preclinical	Curcumin	Natural polylphenol	- Direct interaction (molecular docking analysis) - *FXR* gene induction by Nrf2 pathway	- Attenuates ethanol-induced hepatotoxicity and lipid accumulation in hepatocytes - Protects against ANIT-induced cholestasis in mice - Attenuates hepatic steatosis in high-fat and high-fructose diet-fed mice	[64,144,145]
Preclinical	Silymarin	Natural flavonoid	Direct interaction (molecular docking analysis)	- Ameliorate insulin resistance, dyslipidemia and inflammation, and reconstitutes the BA pool in liver of HFD-induced obesity in mice	[146]
Preclinical	Hedragonic acid	Natural triterpene	Direct interaction (molecular docking analysis)	- Protects against acetaminophen-induced liver injury and inflammation in mice	[147]
Preclinical	Dihydro-artemisinin	Active metabolite of artemisinin compounds	FXR gene induction (mRNA and protein)	- Restricts HSC contraction - Counteracts fibrotic portal hypertension in CCl_4_-treated rats - Protects against alcoholic liver injury and hepatic steatosis in rats	[148,149,150]
Preclinical	Altenusin	Nonsteroidal microbial metabolite	Ligand binding activity of FXR using GAL4-hFXR-LBD (but not PPARα/β/γ, LXRα/β, RXR, ERα, GR, RARα/β/γ, RORα/β/γ)	- Protects against HFD-induced obesity, hyperglycemia, and hepatic steatosis in mice	[151]
Preclinical /Clinical	PX20606 (PX-102)	Nonsteroidal synthetic	Selective FXR agonist	- Induces high-density lipoprotein-mediated transhepatic cholesterol efflux in mice and monkeys - Ameliorates portal hypertension by reducing liver fibrosis, vascular remodeling and sinusoidal dysfunction in CCl_4_-treated rats - Phase I studies (NCT01998659, NCT01998672)	[152,153]
Preclinical	Tropifexor (LJN452)	Nonsteroidal synthetic	Selective FXR agonist	- Induces FXR target genes in the liver and ileum (e.g., SHP, BSEP, and FGF15), and reduces serum triglycerides in rats	[154]
Preclinical	BAR704	Steroidal synthetic	Selective FXR agonist	- Reduces liver fibrosis by interfering with the TGF-Smad3 pathway in HSCs	[155]
Preclinical	BAR502	Non-BA steroidal synthetic	Dual agonist of FXR and TGR5	- Promotes browning of white adipose tissue and reverses liver steatosis and fibrosis in mice fed HFD and fructose	[156]

**Table 2:** Novel candidate compounds for FXR antagonism/inhibition. db/db—leptin receptor-deficient diabetic; HFD—high-fat diet; STZ—streptozotocin; T2DM—type 2 diabetes mellitus.

Study Type	Compound	Chemical Properties	Proposed Mode of Actions (MOAs) for FXR Modulation	Biological Effects Potentially through FXR Modulation	References
Preclinical	Compound-T0	Nonsteroidal synthetic	FXR antagonist	- Increases plasma level of non-high-density lipoprotein cholesterol in mice	[157]
Preclinical	HS218	Nonsteroidal synthetic	FXR antagonist	- Suppresses gluconeogenesis in mouse primary hepatocytes, and improves glucose homeostasis in db/db and HFD/STZ-induced T2DM mice	[158]

## Abbreviations

ABC	ATP-binding cassette
ALD	alcoholic liver disease
AMPK	AMP-activated protein kinase
Bas	bile acids
BDL	bile duct ligation
BSEP	bile salt export pump
CAR	constitutive androstane receptor
CCl_4_	carbon tetrachloride
C/EBP	CCAAT-enhancer-binding protein
CYP7A1	cholesterol 7α-hydroxylase
DILI	drug-induced liver injury
ECM	extracellular matrix
ER	endoplasmic reticulum
FGF	fibroblast growth factor
FGFR4	FGF receptor 4
FXR	farnesoid X receptor
FOXO3a	Forkhead box O3a
GLP1	glucagon-like peptide-1
GSIS	glucose-stimulated insulin secretion
HBx	hepatitis b virus X protein
HCC	hepatocellular carcinoma
HFD	high-fat diet
HNF	hepatocyte nuclear factor
HSCs	hepatic stellate cells
I/R	ischemia-reperfusion
IFNg	interferon gamma
KO	knockout
LXR	live X receptor
MCD	methionine and choline-deficient
MRP	multidrug resistance-associated protein
NAD	nicotinamide adenine dinucleotide
NAFLD	nonalcoholic fatty liver disease
NASH	non-alcoholic steatohepatitis
NLRP3	NACHT, LRR and PYD domains-containing protein 3
NR	nuclear receptor
Nrf2	nuclear factor erythroid-derived 2-related factor 2
NSCLC	non-small cell lung cancer
OATP1B1	organic anion transporting polypeptide 1B1
OCA	obeticholic acid
OST	organic solute transporter
PBC	primary biliary cirrhosis
PDK4	pyruvate dehydrogenate kinase 4
PPAR	peroxisome proliferator-activated receptor
PXR	pregnane X receptor
RXR	retinoid X receptor
SHP	small heterodimer partner
SIRT1	silent information regulator 1
SREBP-1c	sterol response element binding protein-1c
STAT3	signal transducer and activator of transcription 3
TGF	transforming growth factor
TGR5	G protein-coupled BA receptor-1/Takeda G protein-coupled receptor-5
TLR4	Toll-like receptor 4
UDCA	ursodeoxycholic acid
WAT	white adipose tissue
6-ECDCA	6-ethyl-chenodeoxycholic acid
